# Oxytocin Manipulation Alters Neural Activity in Response to Social Stimuli in Eusocial Naked Mole-Rats

**DOI:** 10.3389/fnbeh.2018.00272

**Published:** 2018-11-20

**Authors:** Mariela Faykoo-Martinez, Skyler J. Mooney, Melissa M. Holmes

**Affiliations:** ^1^Department of Cell and Systems Biology, University of Toronto, Toronto, ON, Canada; ^2^Department of Psychology, University of Toronto, Toronto, ON, Canada; ^3^Department of Ecology and Evolutionary Biology, University of Toronto, Toronto, ON, Canada

**Keywords:** social decision-making network, oxytocin, naked mole-rat, eusociality, immediate early gene, social behavior

## Abstract

The social decision-making network (SDMN) is a conserved neural circuit that modulates a range of social behaviors via context-specific patterns of activation that may be controlled in part by oxytocinergic signaling. We have previously characterized oxytocin’s (OT) influence on prosociality in the naked mole-rat, a eusocial mammalian species, and its altered neural distribution between animals of differing social status. Here, we asked two questions: (1) do patterns of activation in the SDMN vary by social context and (2) is functional connectivity of the SDMN altered by OT manipulation? Adult subordinate naked mole-rats were exposed to one of three types of stimuli (three behavioral paradigms: familiar adult conspecific, unfamiliar adult conspecific, or familiar pups) while manipulating OT (three manipulations: saline, OT, or OT antagonist). Immediate early gene c-Fos activity was quantified using immunohistochemistry across SDMN regions. Network analyses indicated that the SDMN is conserved in naked mole-rats and functions in a context-dependent manner. Specific brain regions were recruited with each behavioral paradigm suggesting a role for the nucleus accumbens in social valence and sociosexual interaction, the prefrontal cortex in assessing/establishing social dominance, and the hippocampus in pup recognition. Furthermore, while OT manipulation was generally disruptive to coordinated neural activity, the specific effects were context-dependent supporting the hypothesis that oxytocinergic signaling promotes context appropriate social behaviors by modulating co-ordinated activity of the SDMN.

## Introduction

The social decision-making network (SDMN) is a highly conserved interconnected group of brain regions controlling behaviors related to sex, social dominance, parenting, and affiliation across vertebrates (O’Connell and Hofmann, 2011, 2012). Originally, the network was described by linking the social behavior network (adjacent tegmentum, AH, BNST, MeA, LS, PG, PO, and VMH) and the mesolimbic reward circuit (BLA, BNST, caudate, hippocampus, LS, NAcc, VP, and VTA) (Newman, 1999; O’Connell and Hofmann, 2011). A case can also be made for affiliated nodes such as the olfactory regions (AON, MOB, and Tu) and the medial prefrontal cortex (ACC, CG, IL, and PrL) due to their role in social cognition (Brennan and Kendrick, 2006; Tobin et al., 2010; Lee and Harris, 2013; Nakajima et al., 2014; Bicks et al., 2015; Toor et al., 2015; Oettl et al., 2016; Williamson et al., 2018). Collectively, these regions interact to coordinate incoming social information with context appropriate social responses. Indeed, context dependent behavioral plasticity is likely attributed to changes in coordinated neural activity between nodes of the network, rather than to differential activity of individual brain regions *per se* (Goodson and Kabelik, 2009; Teles et al., 2015; Johnson et al., 2016).

Function of the SDMN is influenced by oxytocin (OT). OT is a neuropeptide implicated in both social (e.g., maternal care, affiliation, and stress) and sexual (e.g., arousal, ejaculation, and motivation) behaviors across vertebrate species (O’Connell and Hofmann, 2012; Anacker and Beery, 2013). For example, manipulation of OT signaling alters multiple social behaviors including prosociality/aggression, social recognition, short-term social memory, alloparenting, and pup care (Ferguson et al., 2000, 2001; Francis et al., 2000; Champagne et al., 2001; Consiglio et al., 2005; Olazábal and Young, 2006a,b; Choleris et al., 2007; Beery et al., 2008; Reddon et al., 2014; Chang et al., 2015). Furthermore, variability in distribution patterns of OT receptors in SDMN regions suggests that oxytocinergic signaling contributes to species-specific adaptations in social behavior (reviewed in Anacker and Beery, 2013). Finally, both central and site-specific (NAcc) manipulation of OT receptor signaling disrupts coordinated activity among SDMN regions (Johnson et al., 2016, 2017). Thus, OT is a key mechanism for sculpting social behavior within and between species, prospectively through facilitating context-specific changes in coordinated activity between nodes of the SDMN.

The naked mole-rat (*Heterocephalus glaber*) exhibits the most extreme form of sociality known in mammals: eusociality. These small, approximately mouse-sized, rodents live in large colonies of up to ∼300 animals with reproduction restricted to one breeding female, the queen, and one to three males (Brett, 1991b; Lacey and Sherman, 1991). The rest of the colony consists of non-breeding subordinates of varying age, which are highly social toward members of their own colony. In contrast, naked mole-rats can be very xenophobic and highly aggressive toward intruding members of another colony (Lacey and Sherman, 1991). Colony members engage in diverse behaviors such as foraging and food-sharing, cooperative care of pups, vocal communication, communal huddling, and colony maintenance and defense (Withers and Jarvis, 1980; Jarvis, 1981; Brett, 1991a; Pepper et al., 1991). There is individual variability in performance of these behaviors, resulting in stable yet plastic task specialization (Jarvis, 1981; Lacey and Sherman, 1991; Mooney et al., 2015b). Subordinates have been further split into two subcastes: workers and soldiers, responsible for colony maintenance and colony defense, respectively (Jarvis, 1981; Lacey and Sherman, 1991; Mooney et al., 2015b).

The oxytocinergic system contributes to the remarkable sociality found in naked mole-rats. Subordinates of both sexes have more OT neurons than breeders in the PVN (Mooney and Holmes, 2013) and peripheral administration of OT to subordinates increases prosocial behaviors in-colony (huddling) and during unfamiliar conspecific interaction tests (proximity, investigation) (Mooney et al., 2014). Among subordinates, workers have higher levels of c-Fos/OT immunoreactive neurons than soldiers in the PVN, accompanied with lower levels of aggression, further suggesting OT promotes prosocial behavior in workers (Hathaway et al., 2016). Naked mole-rats also express OT receptors in the SDMN. They have more OT receptors in the CeA, MeA, BNST, and NAcc in comparison to solitary cape mole-rats, suggesting that OT action in these regions is associated with colonial living (Kalamatianos et al., 2010). Furthermore, OT receptor density varies within naked mole-rats with breeding males showing higher binding than breeding females in the NAcc and males overall showing higher binding than females in the MeA (Kalamatianos et al., 2010; Mooney et al., 2015a).

We hypothesize that OT signaling mediates coordinated neural activity within nodes of the SDMN to promote context-specific social behavior in naked mole-rats. To test this, we had two experimental goals: (1) to determine if coordinated neural activity in the SDMN varies according to social context and (2) to examine if activity of SDMN regions is altered by central OT manipulation. To achieve these goals, we exposed subordinate naked mole-rats of both sexes to three unique social stimuli: a familiar adult conspecific, an unfamiliar adult conspecific, or 1-week-old pups from their home colony while treating with either saline, OT, or an OT antagonist (OTA). Following social exposure, we used immunohistochemistry to stain for the immediate early gene c-Fos to assess activation of brain regions in the SDMN. We predicted that coordinated c-Fos expression within the SDMN regions would differ by social context, and that manipulation of central OT receptor signaling would disrupt these patterns of connectivity.

## Materials and Methods

### Animals and Housing

**FIGURE 1:**
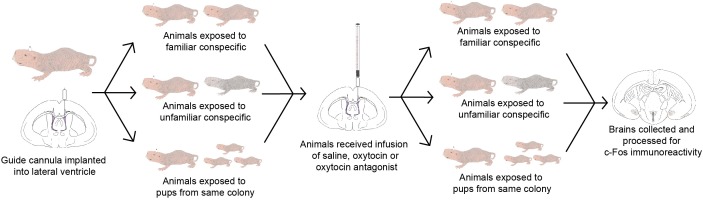
Timeline of the experimental workflow. Animals were implanted with a guide cannula and given 7 days for recovery. On the 8th day, animals were exposed to either a familiar conspecific (*n* = 21), an unfamiliar conspecific (*n* = 30), or 3 pups from the same colony (*n* = 21). On the 9th day, animals were given an infusion of saline, OT, or OTA, and again exposed to either a familiar conspecific (*n* = 7 per OT manipulation), unfamiliar conspecific (*n* = 10 per OT manipulation), or 3 pups from the same colony (*n* = 7 per OT manipulation). Brains were collected 90 min following exposure to stimuli and processed for c-Fos immunoreactivity.

A total of 75 adult subordinate naked mole-rats were used (32 females and 43 males). Animals were considered adults if they were both over 20 g in weight and over 1-year old. Animals weighed between 22 and 60 g were housed on a 12:12 light/dark cycle at 28–30°C and given *ad libitum* access to sweet potato and a wet 19% protein mash (Harlan Laboratories, Inc.). Animals lived in colonies comprised of large (45.75 cm L × 24 cm W × 15.25 cm H) and small (30 cm L × 18 cm W × 13 cm H) polycarbonate cages connected by plastic tubes (25 cm L × 5 cm D). Animals were collected from one of nine colonies ranging in size from 19 to 49 individuals. All procedures adhered to federal and institutional guidelines and were approved by the University Animal Care Committee. A summary of the experimental workflow is presented in Figure 1.

### Intracerebroventricular Cannulation

Experimental animals were implanted with intracerebroventricular cannulae targeting the lateral ventricle as previously described (Mooney and Holmes, 2015). Briefly, mole-rats were deeply anesthetized using isoflurane (induction: 3%, delivered at a rate of 1 L/min inhalation; maintenance: 2%, delivered at a rate of 1 L/min) and the surgical site was cleaned and sterilized with 70% EtOH and then 10% iodine solution (Betadine; repeated twice). Animals were positioned in a stereotaxic instrument (Benchmark^TM^, MyNeurolab.com, St. Louis, MO, United States) and a 1.5 cm incision was made on the top of the head. The skin and muscle were shifted to reveal the skull, which was then cleaned with sterile saline and dried. A small hole was drilled in the skull 0.9 mm lateral and 1 mm anterior to bregma on the right side of the animal and a 22-gauge stainless-steel guide cannula with a 2 mm pedestal (Plastics One, Roanoke, VA, United States) was lowered to 3 mm below the top of the skull. Cyanoacrylate gel was applied to the base of the pedestal to secure the guide cannula to the top of the skull. A dummy cannula was inserted in the guide cannula to prevent exposure, infection, or occlusion. The muscle was then laid back on the skull around the cannula and the skin was sutured together over the pedestal of the guide cannula. A small dab of cyanoacrylate gel was placed on the outside of the dummy cannula at the juncture where the pedestal of the guide cannula and the cap of the dummy cannula meet. Ten minutes prior to the completion of surgery, animals were injected with ketoprofen (Anafen^®^, Merial; 5 mg/kg BW). This injection was also administered once a day for 3 days post-surgery. Animals were placed on top of a heating pad in a cage with clean bedding for 8 h for recovery before being returned to their home colonies.

### Social Behavior Paradigms

#### Familiar Conspecific Interaction Tests

Twenty-one experimental animals (FAM; 9 females and 12 males) were removed from their colony and individually placed in a clear polycarbonate cage (L 43 × W 22 × H 21 cm) lined with corncob bedding. After a 20-min habituation period, an adult conspecific from the experimental animal’s home colony (same-sex pairing, *N* = 12, opposite-sex pairing, *N* = 10; sex matching randomly assigned) was placed in the cage in the corner farthest away from each experimental animal’s current position. Each animal’s behavior was recorded for 20 min with a Sony Handycam^®^. All animals were then returned to their home colony. Twenty-four hours after the baseline test, animals were again removed from their home colony and placed in a clear polycarbonate cage for 10 min before receiving intracerebroventricular infusions of either OT, an OTA, or saline. Seven animals received 0.25 μg of OT (OT acetate salt hydrate or α-hypophamine, No. O6379; Sigma), seven animals received 1 ng of the specific OTA (d(CH2)51,Tyr(Me)2,Thr4,Orn8,des-Gly-NH29)-Vasotocin, No. H-2908 BACHEM), and seven animals received 1 μl of the sterile saline. These doses were chosen as they produce behavioral effects in similarly sized mammals (Ferguson et al., 2000; Shahrokh et al., 2010; Samuelsen and Meredith, 2011). For infusion, animals were lightly anesthetized with isoflurane (2% delivered at a rate of 1 L/min) and the dummy cannula was replaced with an internal infusion cannula that sat 0.1 mm below the base of the guide cannula. One microliter of the drug or saline was infused via 500-μl Bas gas-tight syringes (MD-0050; Bio Analytical Systems) connected to the internal cannula with PE50 tubing. Infusions were automated at a rate of 1 μl/min with a Harvard infusion pump (Harvard Apparatus Inc. 22, Natick, MA, United States). Animals were then removed from anesthesia and recovered in ∼2–3 min. After 10 additional minutes, a novel stimulus animal was introduced following the same procedure as for the baseline test. Target behaviors for the baseline test and the manipulation test (Table 1) were scored by an observer blind to the experimental condition using Observer XT software (Noldus).

**Table 1:** Behaviors of interest.

Behavior	Description
Aggression	Duration of physical attack on stimulus and stand-off with stimulus with teeth barred
Anogenital investigation	Duration of direct sniffing of the stimulus animal in anogenital region (sexual behavior)
Investigation (flank/face)	Duration of direct sniffing of the stimulus animal (aggressive)
Pup carrying	Duration of time with pup between the experimental animal’s incisors
Pup investigation	Duration of time with the experimental animal’s snout directed toward pup but without pup in the animal’s incisors

*Aggression, anogenital investigation, and investigation (flank/face) were scored in animals interacting with a familiar or unfamiliar conspecific. Pup carrying and pup investigation were scored in animals interacting with pups.*

#### Unfamiliar Conspecific Interaction Tests

Thirty experimental animals (UNFAM; 15 females and 15 males) were used in this paradigm. All procedures and manipulations were identical to the familiar conspecific paradigm described above with the exception of the stimulus animal. In this case, an adult conspecific from an *unfamiliar* colony was used as the stimulus (same-sex pairing, *N* = 12, opposite-sex pairing, *N* = 10; sex matching randomly assigned). Ten animals received OT, 10 animals received OTA, and 10 animals received sterile saline.

#### Pup Interaction Tests

Twenty-four experimental animals (PUP; 8 females and 16 males) were used in this paradigm. Again, procedures and manipulations were identical to the FAM paradigm with the exception of the social stimulus. In this case, three pups (∼1 week old) from the experimental animal’s home colony were placed in the cage in the corner farthest away from each animal’s current position. Eight animals received OT, nine animals received OTA, and seven animals received sterile saline.

### Tissue Collection and c-Fos Immunohistochemistry

One hundred minutes after the start of each interaction test (FAM, UNFAM, PUP) in which OT activity was manipulated, animals were overdosed with avertin (400 mg/kg) and rapidly decapitated. Brains were extracted and post-fixed in 4% paraformaldehyde for 4 h before being transferred to sucrose [30% in phosphate-buffered saline (PBS)] and stored for at least 24 h at 4°C. Brains were sliced coronally at 30 μm into four series on a freezing microtome. One series was stained for c-Fos immunoreactivity. Tissue was washed for 15 min (3 × 5 min) in PBS, followed by a 90-min incubation in blocking solution at room temperature [4% NGS, 0.3% TritonX, 3% H_2_O_2_ (3%) in PBS]. Tissue was then rinsed in PBS for 15 min (3 × 5 min) and incubated in c-Fos primary antibody [1:1200 rabbit anti-c-Fos polyclonal antibody (Santa Cruz Biotechnology) in PBS with 4% NGS, 0.3% TritonX] at 4°C for approximately 24 h. Tissue was rinsed in PBS for 15 min (3 × 5 min) and then incubated for 90 min at room temperature in a secondary antibody solution [1:200 goat anti-rabbit (Vector Laboratories) in PBS with 0.3% TritonX and 2% NGS]. Tissue was rinsed again in PBS for 15 min (3 × 5 min) and then incubated at room temperature for 90 min in avidin–biotin complex (ABC Elite, Vector Laboratories). Sections were washed again for 15 min (3 × 5 min) in PBS, and c-Fos was visualized using nickel-enhanced 3-3′-diaminobenzidine (DAB) for 3 min [2% DAB (1.25%), 0.2% H_2_O_2_ (3%), 0.24% NiCl (8%) in PBS]. Tissue was then mounted onto slides coated in pig gelatin, dehydrated, and coverslipped with Permount (Fisher Scientific). Experimental groups were yoked across staining cohorts.

### c-Fos Quantification

The number of c-Fos immunoreactive cells was counted using either OpenCFU (Beta version 3.9.0) or ImageJ (Rasband, 2012; Geissmann, 2013). For a given brain region, counts for all animals were performed using the same program. As tissue tearing and damage was more likely on the side of the brain ipsilateral to the cannula implantation, counts were done unilaterally on the side contralateral to the implantation. On OpenCFU, this was done automatically with a threshold setting of 5 and a radius setting of 5. Counts were verified by an observer blind to the experimental condition for all sections. Areas counted using OpenCFU were the AH, BLA, BNST, CeA, LS, MeA, MS, NAcc, PG, PO, PVN, SON, and VMH. Using ImageJ, images were converted to 8-bit, a threshold of 180–190 applied, and particles larger than 10 pixels^2^ counted. Areas counted using ImageJ were the ACC, AON, CA1, CA2, CA3, caudate, CG, dDG, IL, MOB, PIC, PrL, Tu, vDG, VP, and VTA. For each region of interest, a photomicrograph was taken on three consecutive slices of tissue using a microscope mounted camera at 200× magnification. The CA1, CA2, CA3, dDG, and vDG were taken at 400× magnification to prevent counting other surrounding regions. Photomicrographs contained the same area for all animals, corresponding to the given region; all c-Fos labeled cells in the photomicrograph were counted. The exceptions to this were the BNST, which only had pictures taken from two consecutive slices because only two sections reliably contained the BNST in each series, and the PAG and VTA, which only had one slice counted because more caudal sections were not collected during slicing.

### Placement Confirmation

Before euthanizing animals, one saline-treated animal from each behavioral paradigm was also infused with 1 μl of india ink (10% v/v) in order to confirm that fluid was diffusing throughout the brain. For all animals, tissue was examined to ensure that the cannula clearly penetrated the lateral ventricle. Because we could not definitively confirm penetration of the ventricles in two FAM animals (one receiving saline and one receiving OTA), two UNFAM animals (both receiving OT), and three PUP animals (one receiving saline, one receiving OT, and one receiving OTA), these animals were excluded from analyses. Final sample sizes are reported in the section “Social behavior paradigms.”

### Statistical Analyses

All statistical analyses were performed on raw counts of c-Fos immunoreactive cells. First, a linear mixed-effects model was run using the nlme and lmerTest packages in R (R Development Core Team, 2011; Kuznetsova et al., 2016; Pinheiro et al., 2016). c-Fos immunoreactivity was the response variable and behavioral paradigm, OT manipulation, brain region, and sex were predictor variables, using animal ID and immunohistochemistry batch as random effects. The linear mixed-effects model was used to determine if any predictor variables could be collapsed for the rest of the analyses; due to non-significant effects, sex was dropped as a variable for subsequent analyses. We then performed brain region-specific linear mixed-effects models with c-Fos as the response variable, behavioral paradigm, and OT manipulation as predictor variables, and batch as a random effect, again using the nlme and lmerTest packages in R. A Bonferroni correction was used to adjust for multiple testing (30 tests = *p*-value set at 0.0017). Main effects reaching this criterion (*p* < 0.0017) were followed with Tukey’s HSD *post hoc* tests.

We next explored coordinated activity between brain regions by examining correlations in c-Fos expression across all brain regions measured for each behavioral paradigm-by-OT manipulation group. The Hmisc package in R was used to perform pair-wise Pearson correlations for all brain regions, followed by visualization using the corrplot package (Wei and Simko, 2016; Harrell, 2017). No thresholding was applied, but significant correlations are marked by white asterisks on the plots. To further explore the effect of behavioral paradigm and OT manipulation on how c-Fos is expressed between regions, networks were produced for each behavioral paradigm-by-OT manipulation group. For the networks, correlation *p*-values were corrected using the Benjamini–Hochberg procedure at a 5% false-discovery rate. These correlations were then extracted and plotted using Cytoscape (Shannon et al., 2003). Cytoscape’s built-in Network Analyzer tool was used to plot node size according to degree (how many edges correspond to a given node) and edges according to correlation strength. On the networks, nodes are clustered and color-coded based on anatomy and literature-based functions into the following: olfactory (AON, MOB, PIC, Tu), mPFC (ACC, CG, IL, PrL), social behavior network (AH, BNST, LS, MS, PG, PO, VMH), amygdala (BLA, CeA, MeA), reward (caudate, NAcc, VP, VTA), OT production (AntPVN, PostPVN, SON), and hippocampal (CA1, CA2, CA3, dDG, vDG).

Finally, to examine if OT manipulation affected behavior, we tested two linear mixed-effects models as described above. First, to test whether injection manipulation itself affected behavior (because animals were anesthetized for ICV injections), we modeled difference in behavior duration between baseline and test day as the response variable, treatment and sex as independent variables, and animal ID as the random effect variable. Second, to test whether behavior on the test day was altered by OT manipulation, we modeled duration of behavior on test day as the response variable, treatment and sex as independent variables, and animal ID as the random effect variable. Both models were repeated for each behavior tested per behavioral paradigm and corrected for multiple testing using the Bonferroni method (FAM/UNFAM: three comparisons = *p*-value < 0.017; PUP: eight comparisons = *p*-value < 0.0062).

Then, to determine if c-Fos expression is related to behavior, we clustered brain regions using factor analysis to reduce analyses performed: all saline-treated animals across paradigms were included using the dimension reduction function in SPSS (IBM Corp, 2016). Principal axis factoring with a Promax rotation was used for the unsupervised clustering of brain regions; four clusters (as listed in the section “Results”) were produced. Next, for each paradigm (collapsed across OT manipulation), a Pearson correlation between the summed c-Fos counts for a given cluster and given behavior on test day was performed. For FAM and UNFAM animals, duration of anogenital investigation, face/flank investigation, and aggression was scored. For PUP animals, duration of pup carrying and pup interaction was scored. *P*-values were corrected for multiple testing using the Bonferroni method (FAM/UNFAM: 12 comparisons = *p*-value < 0.0042; PUP: 8 comparisons = *p*-value < 0.0062).

## Results

### Brain Region-Specific Analyses Reveal OT Manipulation and Paradigm Main Effects

The LME including all brain regions revealed a significant main effect of behavioral paradigm [*F*(2,57) = 4.8065, *p* = 0.0118], OT manipulation [*F*(2,57) = 6.3197, *p* = 0.0033], and brain region [*F*(29,1608) = 108.4381, *p* < 0.0001], but not sex [*F*(1,57) = 1.0054, *p* = 0.3203]. Significant interactions were detected for behavioral paradigm-by-brain region [*F*(58,1608) = 4.7031, *p* < 0.0001] and OT manipulation-by-brain region [*F*(58,1608) = 2.1290, *p* < 0.0001], but not for sex-by-brain region [*F*(29,1608) = 0.3893, *p* = 0.9986].

**FIGURE 2:**
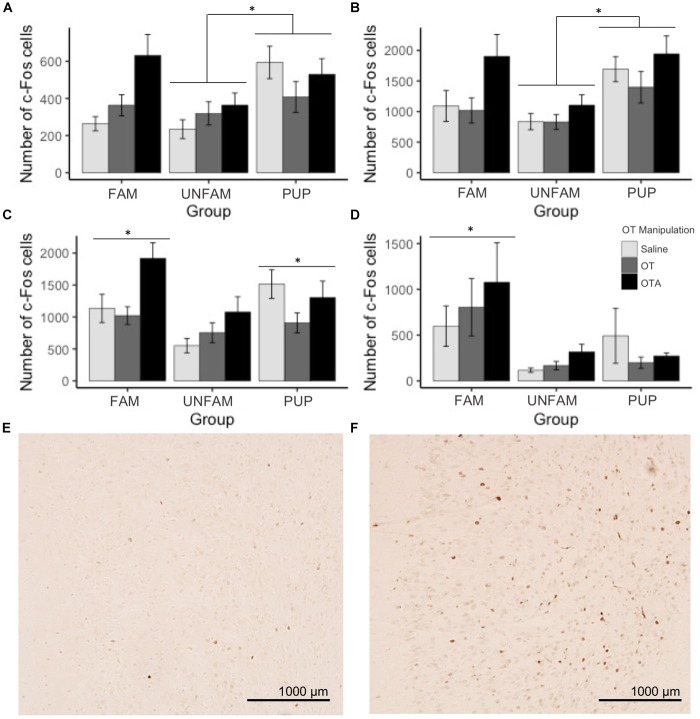
Number of c-Fos cells ± SEM in brain regions where expression significantly varied by behavioral paradigm. UNFAM animals had a significantly lower number of c-Fos cells in the **(A)** MOB (main effect: *p* = 0.0011) relative to PUP (*p* = 0.002) but not FAM (*p* = 0.42), **(B)** Tu (main effect: *p* < 0.0001) relative to PUP (*p* = 0.00012) and FAM (*p* = 0.059), and **(C)** IL (main effect: *p* = 0.0015) relative to FAM (*p* = 0.002) and PUP (*p* = 0.018). **(D)** FAM animals had a significantly higher number of c-Fos cells in the VP (main effect: *p* = 0002) relative to UNFAM (*p* = 0.0003) and PUP (*p* = 0.006). Photomicrographs showing the reduced c-Fos immunoreactivity in the IL of an UNFAM animal **(E)** compared to the IL of a **(F)** FAM animal. IL, infralimbic cortex; Tu, olfactory tubercle; VP, ventral pallidum; UNFAM, animals exposed to an unfamiliar conspecific; FAM, animals exposed to a familiar conspecific.

**FIGURE 3:**
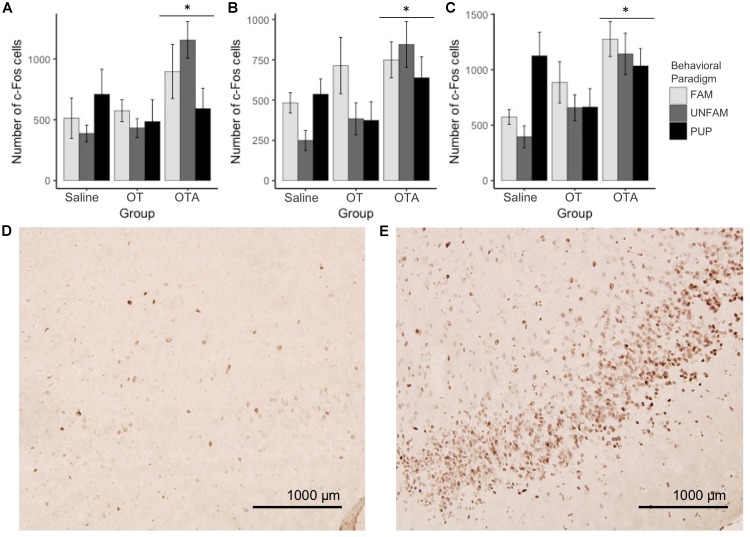
Number of c-Fos cells ± SEM in brain regions where expression significantly varied by OT manipulation. OTA-treated animals had a significantly higher number of c-Fos cells in the **(A)** PIC (*p* = 0.00167), **(B)** ACC (*p* = 0.0014), and **(C)** PrL (*p* = 0.000862). Photomicrographs showing the reduced c-Fos immunoreactivity in the PIC of a **(D)** saline-treated animal compared to the PIC of an **(E)** OTA-treated animal. PIC, piriform cortex; ACC, anterior cingulate cortex; PrL, pre-limbic cortex.

For brain region-specific analyses, only results that were statistically significant after correcting for multiple testing (Bonferroni method) are reported here (*p* < 0.0017). All effects with *p* < 0.05 are shown in Table 2 while raw data are available in the Supplementary Table 1. A main effect of behavioral paradigm [*F*(2,61) = 7.61, *p* = 0.0011; Figure 2A] revealed altered c-Fos expression in the MOB, with PUP animals having higher expression of c-Fos relative to UNFAM animals (*p* = 0.002). This same pattern was seen in the Tu [*F*(2,61 = 10.9, *p* < 0.0001; Figure 2B], with a significant difference between PUP and UNFAM animals (*p* = 0.00012) and a trend between FAM and UNFAM animals (*p* = 0.059). In the IL, a main effect of behavioral paradigm [*F*(2,61) = 7.3, *p* = 0.0015; Figure 2C] revealed reduced c-Fos in UNFAM animals relative to both FAM and PUP animals whereas in the VP [*F*(2,63), *p* = 0.0002; Figure 2D], c-Fos was higher in FAM animals relative to both UNFAM and PUP groups. Main effects of OT manipulation revealed higher c-Fos expression in OTA-treated animals relative to both saline- and OT-treated animals in the PIC [*F*(2,63) = 6.9, *p* = 0.00167; Figure 3A], ACC [*F*(2,61) = 7.4, *p* = 0.0014; Figure 3B], and PrL [*F*(2,61) = 8.3, *p* = 0.000862; Figure 3C].

**Table 2:** Brain region-specific linear mixed effect model results listed by brain region with significance value (Sig) and direction of effect (Dir).

	Region	Main effect of behavioral paradigm	Main effect of OT manipulation	Behavioral paradigm-by-OT manipulation interaction
AON	Sig. Dir.	*F*(2,61) = 3.55, *p* = 0.0346 PUP > UNFAM^∗^		
MOB	Sig. Dir.	*F*(2,61) = 7.61, *p* = 0.0011 PUP > UNFAM^∗^		
Tu	Sig. Dir.	*F*(2,61) = 10.95, *p* = 0.0001 FAM/PUP^∗^ > UNFAM	*F*(2,61) = 5.86, *p* = 0.005 OTA > Saline OTA > OT^∗^	
PIC	Sig. Dir.		*F*(2,63) = 6.94, *p* = 0.002 OTA > Saline/OT^∗^	
ACC	Sig. Dir.		*F*(2,63) = 7.37, *p* = 0.001 OTA > Saline^∗^	
IL	Sig. Dir.	*F*(2,61) = 7.29, *p* = 0.0015 FAM/PUP > UNFAM^∗^	*F*(2,61) = 5/64, *p* = 0.006 OTA > OT^∗^/Saline	
PrL	Sig. Dir.		*F*(2,61) = 8.32, *p* = 0.0006 OTA > Saline/OT^∗^	*F*(2,61) = 2.63, *p* = 0.043 PUP Saline/FAM OTA/PUP OTA/UNFAM OTA > UNFAM Saline^∗^
CG	Sig. Dir.			*F*(4,58) = 4.38, *p* = 0.004 FAM OT > UNFAM Saline FAM OT^∗^/UNFAM OTA > PUP OTA
PO	Sig. Dir.	*F*(2,63) = 4.72, *p* = 0.012 FAM > UNFAM^∗^/PUP		
AH	Sig. Dir.	*F*(2,63) = 3.87, *p* = 0.026 FAM > UNFAM^∗^/PUP		
VMH	Sig. Dir.	*F*(2,63) = 6.41, *p* = 0.003 FAM > UNFAM^∗^/PUP	*F*(2,63) = 3.44, *p* = 0.038 OTA > OT^∗^/Saline	
PG	Sig. Dir.	*F*(2,63) = 4.01, *p* = 0.023 FAM > UNFAM^∗^	*F*(2,63) = 4.70, *p* = 0.012 OTA > OT^∗^/Saline	
BNST	Sig. Dir.	*F*(2,63) = 3.81, *p* = 0.027 FAM > UNFAM^∗^		
LS				
MS				
AntPVN				
PostPVN				
SON	Sig. Dir.	*F*(2,63) = 6.67, *p* = 0.002 FAM > UNFAM^∗^/PUP^∗^	*F*(2,61) = 6.80, *p* = 0.002 OTA > Saline^∗^ OT > Saline	*F*(4,63) = 3.83, *p* = 0.008 FAM OT/FAM OTA/PUP Saline/PUP OT/Saline/PUP OTA/UNFAM OTA > UNFAM Saline^∗^ UNFAM OTA > UNFAM OT
MeA	Sig. Dir.		*F*(2,61) = 4.83, *p* = 0.011 OTA > Saline/OT^∗^	
BLA	Sig. Dir.		*F*(2,63) = 3.55, *p* = 0.035 OTA > OT^∗^	
CEA	Sig. Dir.	*F*(2,63) = 3.15, *p* = 0.050 FAM > UNFAM^∗^/PUP	*F*(2,63) = 3.53, *p* = 0.035 OTA > Saline^∗^/OT	
Caudate				
VP	Sig. Dir.	*F*(2,63) = 9.57, *p* = 0.0002 FAM > UNFAM/PUP^∗^		
NAcc	Sig. Dir.		*F*(2,63) = 3.60, *p* = 0.033 OTA > OT/Saline	
VTA				
CA1				
CA2				
CA3				
dDG				
vDG	Sig. Dir.	*F*(2,57) = 3.42, *p* = 0.039 FAM > UNFAM^∗^	*F*(2,57) = 4.34, *p* = 0.018 OTA > OT^∗^	*F*(4,57) = 3.67, *p* = 0.010 PUP Saline > PUP OT^∗^/UNFAM OT FAM OT^∗^/FAM OTA^∗^/UNFAM OTA > PUP OT FAM OTA > UNFAM OT^∗^

*All main effects with p < 0.05 are shown; however, only those with p < 0.0017 are considered significant following Bonferroni multiple testing correction. Significant post hoc effects (p < 0.05) are indicated with an asterisk (^∗^). AON, anterior olfactory nucleus; MOB, main olfactory bulb; Tu, olfactory tubercle; PIC, piriform cortex; ACC, anterior cingulate cortex; IL, infralimbic cortex; PrL, pre-limbic cortex; CG, cingulate cortex; PO, pre-optic area; AH, anterior hypothalamus; VMH, ventromedial hypothalamus; PG, periaqueductal gray; BNST, bed nucleus of the stria terminalis; LS, lateral septum; MS, medial septum; AntPVN, anterior paraventricular nucleus; PostPVN, posterior paraventricular nucleus; SON, supraoptic nucleus; MeA, medial amygdala; BLA, basolateral amygdala; CeA, central amygdala; Caudate, caudate putamen; VP, ventral pallidum; NAcc, nucleus accumbens; VTA, ventral tegmental area; CA1, cornu ammonis 1; CA2, cornu ammonis 2; CA3, cornu ammonis 3; dDG, dorsal dentate gyrus; vDG, ventral dentate gyrus; UNFAM, animals exposed to unfamiliar conspecific; FAM, animals exposed to familiar conspecific; PUP, animals exposed to pups; OT, oxytocin; OTA, oxytocin antagonist.*

### Correlation Matrix Plots and Network Visualization Reveal the Disruption of SDMN Pathways by OT Manipulation

**FIGURE 4:**
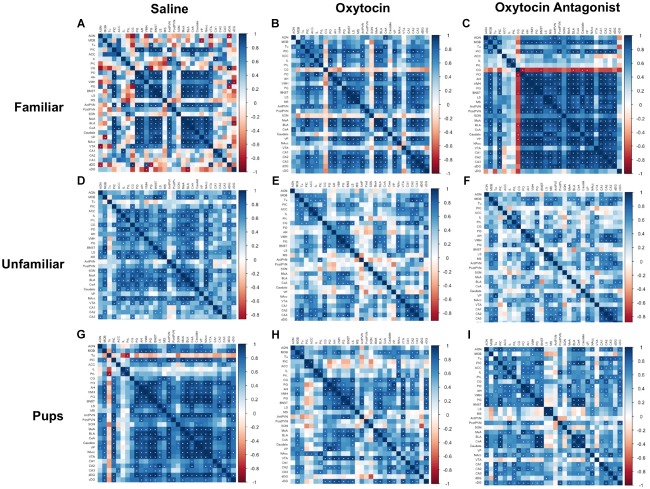
Correlation matrices separated by behavioral paradigm and OT manipulation. Pair-wise Pearson correlations were calculated for c-Fos cell counts and all correlations plotted. Statistically significant correlations are marked by a white asterisk. **(A)** Saline-treated FAM, **(B)** OT-treated FAM, **(C)** OTA-treated FAM, **(D)** saline-treated UNFAM, **(E)** OT-treated UNFAM, **(F)** OTA-treated UNFAM, **(G)** saline-treated PUP, **(H)** OT-treated PUP, and **(I)** OTA-treated PUP. AON, anterior olfactory nucleus; MOB, main olfactory bulb; Tu, olfactory tubercle; PIC, piriform cortex; ACC, anterior cingulate cortex; PrL, pre-limbic cortex; IL, infralimbic cortex; CG, cingulate cortex; PO, pre-optic area; AH, anterior hypothalamus; VMH, ventromedial hypothalamus; PG, periaqueductal gray; BNST, bed nucleus of the stria terminalis; LS, lateral septum; MS, medial septum; AntPVN, anterior paraventricular nucleus; PostPVN, posterior paraventricular nucleus; SON, supraoptic nucleus; MeA, medial amygdala; BLA, basolateral amygdala; CeA, central amygdala; Caudate, caudate putamen; VP, ventral pallidum; NAcc, nucleus accumbens; VTA, ventral tegmental area; CA1, cornu ammonis 1; CA2, cornu ammonis 2; CA3, cornu ammonis 3; dDG, dorsal dentate gyrus; vDG = ventral dentate gyrus.

The correlation matrices were performed within each behavioral paradigm-by-OT manipulation group (Figure 4). Regions were clustered and color-coded based on literature and known functional connections in order to better visualize relationships between related regions. The plots demonstrate that manipulation of OT signaling, either by OT or OTA treatment, alters correlated activity among SDMN regions. In saline-treated FAM animals (Figure 4A), significant correlations are largely restricted to core social behavior network and reward-related regions. While OT (Figure 4B) had modest effects on c-Fos expression patterns, OTA (Figure 4C) caused a striking increase in positive correlations between almost all brain regions. In saline-treated UNFAM animals (Figure 4D), the SDMN-related regions are active and positively correlated. Treatment with OT (Figure 4E) or OTA (Figure 4F) disrupts these positive correlations. The saline-treated PUP animals (Figure 4G) are similar to UNFAM animals in that brain regions are significantly, positively correlated to one another and this pattern is disrupted by both OT (Figure 4H) and OTA (Figure 4I).

**FIGURE 5:**
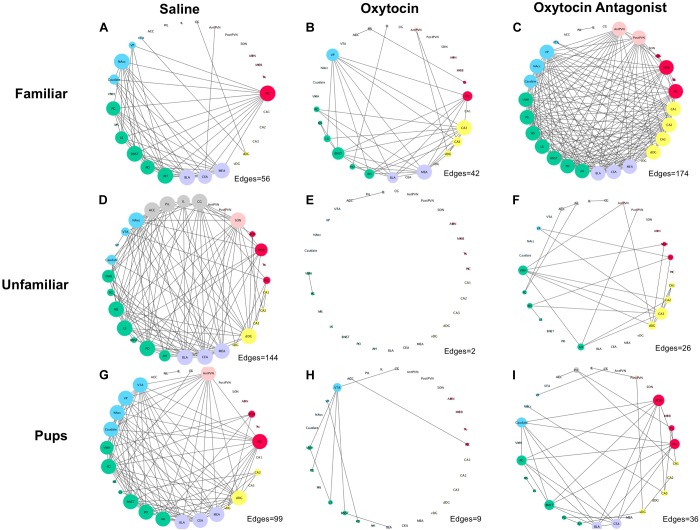
Correlation networks separated by behavioral paradigm and OT manipulation. Pair-wise Pearson correlations were calculated for c-Fos cell counts with multiple-testing corrected for using the Benjamini–Hochberg method with a false discovery rate of 5%. Brain regions are color-coded based on anatomically and functionally similar groups (blue, reward; green, social behavior network; purple, amygdala; yellow, hippocampus; red, olfactory; pink, OT production cluster; and gray, medial prefrontal cortex). Node size is a measure of degree (how many edges are connected to a given node). **(A)** Saline-treated FAM, **(B)** OT-treated FAM, **(C)** OTA-treated FAM, **(D)** saline-treated UNFAM, **(E)** OT-treated UNFAM, **(F)** OTA-treated UNFAM, **(G)** saline-treated PUP, **(H)** OT-treated PUP, and **(I)** OTA-treated PUP. AON, anterior olfactory nucleus; MOB, main olfactory bulb; Tu, olfactory tubercle; PIC, piriform cortex; ACC, anterior cingulate cortex; PrL, pre-limbic cortex; IL, infralimbic cortex; CG, cingulate cortex; PO, pre-optic area; AH, anterior hypothalamus; VMH, ventromedial hypothalamus; PG, periaqueductal gray; BNST, bed nucleus of the stria terminalis; LS, lateral septum; MS, medial septum; AntPVN, anterior paraventricular nucleus; PostPVN, posterior paraventricular nucleus; SON, supraoptic nucleus; MeA, medial amygdala; BLA, basolateral amygdala; CeA, central amygdala; Caudate, caudate putamen; VP, ventral pallidum; NAcc, nucleus accumbens; VTA, ventral tegmental area; CA1, cornu ammonis 1; CA2, cornu ammonis 2; CA3, cornu ammonis 3; dDG, dorsal dentate gyrus; vDG, ventral dentate gyrus.

The networks, like the correlation matrices, are plotted as behavioral paradigm-by-OT manipulation groups; however, unlike the correlation plots only relationships that were significant after applying Benjamini–Hochberg thresholding were included in the networks (5% false discovery rate). The networks confirm that OTA treatment alters correlated activity across the SDMN for all paradigms: by increasing connections in the FAM paradigm and disrupting connections in the UNFAM and PUP paradigms. OT had similar effects as OTA in the UNFAM and PUP paradigms. In the network for FAM saline-treated animals (Figure 5A), the NAcc, PIC, amygdalar regions, and some parts of the social behavior network are key nodes with highly correlated co-expression. OTA (Figure 5C) results in most all regions except the prefrontal cortex regions and SON highly co-expressing, while OT (Figure 5B) results in a more random expression pattern. In the network for UNFAM saline-treated animals (Figure 5D), the SBN, prefrontal cortex, amygdala, NAcc, SON, MOB, and dDG are important nodes. Almost all edges are removed by OT (Figure 5E) treatment while OTA (Figure 5F) activates hippocampal regions but results in few correlations between other regions. Finally, in the network for PUP saline-treated (Figure 5G), there is high co-expression in most of the SBN, amygdala, reward-related regions, PIC, AntPVN, and dDG. OT (Figure 5H) and OTA (Figure 5I) treatment eliminated most of these edges.

### Factor Analysis Reveals Clusters of Related Regions Reflecting Known Patterns

There were no differences in behavior between baseline and test day, indicating that ICV injection did not in and of itself alter behavior. OT manipulation did not significantly affect behavior on test day. A summary of these results can be found in Supplementary Tables 1, 2. Factor analysis revealed four clusters of brain regions (Table 3). Cluster 1 contains most classic SDMN regions (AH, BNST, LS, MeA, MS, PG, PIC, PO, VMH), cluster 2 is prefrontal cortex and OT production-related regions (ACC, AntPVN, CG, IL, PostPVN, PrL, SON), cluster 3 is hippocampal (CA1, CA2, CA3, dDG, MOB, vDG), and cluster 4 is olfactory (AON, Tu). Correlations between the summed counts of c-Fos within each cluster and behaviors revealed no significant relationships between any particular behavior and brain region cluster.

**Table 3:** Factor analysis results following application of principal axis factoring to saline-treated animals (collapsed across task), identifying four clusters of brain regions.

Cluster	Brain regions
Cluster 1	PIC, PO, AH, VMH, PG, BNST, LS, MS, MeA, BLA, CeA, Caudate, VP, NAcc, VTA
Cluster 2	ACC, PrL, IL, CG, AntPVN, PostPVN, SON
Cluster 3	MOB, CA1, CA2, CA3, dDG, vDG
Cluster 4	AON, Tu

*PIC, piriform cortex; PO, pre-optic area; AH, anterior hypothalamus; VMH, ventromedial hypothalamus; PG, periaqueductal gray; BNST, bed nucleus of the stria terminalis; LS, lateral septum; MS, medial septum; MeA, medial amygdala; BLA, basolateral amygdala; CeA, central amygdala; Caudate, caudate putamen; VP, ventral pallidum; NAcc, nucleus accumbens; VTA, ventral tegmental area; ACC, anterior cingulate cortex; PrL, pre-limbic cortex; IL, infralimbic cortex; CG, cingulate cortex; AntPVN, anterior paraventricular nucleus; PostPVN, posterior paraventricular nucleus; SON, supraoptic nucleus; MOB, main olfactory bulb; CA1, cornu ammonis 1; CA2, cornu ammonis 2; CA3, cornu ammonis 3; dDG, dorsal dentate gyrus; vDG, Ventral dentate gyrus; AON, anterior olfactory nucleus; Tu, olfactory tubercle.*

## Discussion

In this study, we investigated the effects of OT manipulation on coordinated activation of brain regions composing and related to the SDMN. By quantifying c-Fos immunoreactive cells following three different social paradigms, we demonstrated that the SDMN is conserved in the highly social naked mole-rat and that it functions in a context-dependent manner. Furthermore, we report that OT manipulation, both via OT and OTA, is generally disruptive to coordinated neural activity, though specific effects are context-dependent. Finally, these data suggest that while structures such as the medial prefrontal cortex, hippocampus, and olfactory regions are important for processing social stimuli, they are not direct members of the SDMN.

By examining neural activity in SDMN regions following different behavioral paradigms, we have demonstrated that, perhaps not surprisingly, this network is functionally conserved in naked mole-rats. This is consistent with previous neurochemical analyses in SDMN regions in this species (Rosen et al., 2007, 2008; O’Connell and Hofmann, 2012; Holmes et al., 2013; Mooney et al., 2015a; Beery et al., 2016). Factor analysis shows the examined brain regions from 4 clusters (Table 3), the first of which includes all the SDMN brain regions together except the hippocampus. The other three clusters comprise anatomically and functionally related regions (i.e., medial prefrontal cortex, hippocampus, and olfactory regions, respectively). This pattern of clustering corroborates the existence of a SDMN in the naked mole-rat, and further suggests the other three clusters are perhaps related, but not directly a part of the SDMN. Interestingly, the SDMN showed correlated c-Fos immunoreactivity in the FAM saline-treated group. Given the colony living of naked mole-rats, their baseline experience is consistent interaction with multiple familiar conspecifics. When Newman proposed the social behavior network, one limitation was that without social stimuli, there is no baseline activity for the associated regions (Newman, 1999; O’Connell and Hofmann, 2011). In the case of eusocial naked mole-rats, it is likely the SDMN is always active and integral for navigating colony life.

The SDMN is thought to modulate behaviors necessary for navigating varied social environments (O’Connell and Hofmann, 2011). The behavioral paradigm-specific patterns of neural activity that we report support the hypothesis that coordinated activation of brain regions within the SDMN is context-dependent. Examination of the correlation matrices (Figure 4) reveals that while saline-treated animals in all three behavioral paradigms show coordinated activation of SDMN regions, the network incorporates the medial prefrontal cortex regions in UNFAM animals and hippocampal regions in PUP animals. The medial prefrontal cortex has been implicated in social approach, social ascent, facilitating aggressive behavior and in establishing social rank in mice, hence activation could be associated with need for assessing status and establishing hierarchy between the two unfamiliar animals (Wang et al., 2011; Takahashi et al., 2014; Lee et al., 2016; Williamson et al., 2018). It is interesting to speculate that the coordinated activation of hippocampal regions in PUP animals reflects learning of new colony members and is consistent with the increase in cell proliferation observed following pup exposure in prairie voles and fatherhood in male California mice, both species that exhibit biparental care (Ruscio et al., 2008; Hyer et al., 2016). Alternatively, this could be related to spatial memory required for returning pups to their nest as seen in female rats (Kinsley et al., 1999). In addition to paradigm-specific patterns of correlated activity, we also report paradigm-specific effects on c-Fos expression in individual brain regions. For example, the VP had significantly higher c-Fos immunoreactivity in FAM animals (Figure 2C). This region is involved in formation of social attachment in socially monogamous prairie voles (Lim and Young, 2004; Barrett et al., 2013; Zheng et al., 2013). In UNFAM animals, who are meeting a novel animal and presumably assessing relative status, there was significantly lower activation of the MOB, Tu, and IL (Figures 2A–C). The olfactory system is essential to pair bonding in prairie voles (Curtis et al., 2001), and, while naked mole-rat subordinates are reproductively inactive, they are highly affiliative with colony members. Naked mole-rats show preference for familiar olfactory cues over unfamiliar ones (Toor et al., 2015), likely contributing to in-colony social recognition and bonding. Interestingly, the IL (in addition to the PrL) is also activated in a shifting social environment in which social ascent is possible, as in the case of removing an alpha male in a mouse hierarchy (Williamson et al., 2018). Administration of a histone deacetylase inhibitor to the IL increases stress behaviors in a Syrian hamster model of social defeat (McCann et al., 2017), further implicating the IL in social interactions involving dominance.

The data reported here also support the hypothesis that OT mediates coordinated neural activity within the SDMN to promote context-specific social behavior. OTA treatment altered coordinated c-Fos expression in all tasks but while it increased connectivity in FAM animals, it reduced connectivity in UNFAM and PUP animals (Figures 4, 5). Brain region-specific analyses revealed that in all behavioral paradigms, OTA significantly increased c-Fos expression in the PIC, ACC, and PrL (Figure 3), suggesting these regions are particularly sensitive to perturbations in OT signaling. While examination of OT receptor distribution in naked mole-rats did not evaluate anterior regions like the ACC and PrL, subordinate NMRs have negligible OT receptor binding in the PIC (Kalamatianos et al., 2010). Thus, OTA could be acting elsewhere to indirectly influence activity in these regions. Similar to OTA, we also found evidence that OT treatment alters connectivity in the SDMN with reduced coordinated activity in animals from the UNFAM and PUP paradigms (Figure 5). This is not necessarily surprising as exogenous OT is unlikely to reflect endogenous levels and, furthermore, our central delivery ensures OT would act at multiple targets, interfering with “normal” OT signaling. Importantly, we cannot infer whether more or fewer connections are optimal as we have yet to directly relate coordinated activity to specific behaviors. Rather, we can conclude that central OT manipulation affects connectivity in the SDMN in various social contexts and future studies will identify how specific regions are involved.

Indeed, the NAcc is a likely hub modulating the role of OT between other SDMN regions. OT acts in the NAcc to modulate sociosexual interactions and mating in prairie voles (Johnson et al., 2017): administration of an OT receptor antagonist directly to the NAcc changes connectivity between NAcc and other SDMN regions. The NAcc is also involved in reproductive behavior and social dominance in naked mole-rats. For example, OT receptor binding is higher in breeding male NAcc compared to breeding females (Mooney et al., 2015a) and expression of genes involved in social suppression of reproduction (*Kiss1*, *Npvf*, *Gpr147*, *Tac3r*) varies by sex and status (Faykoo-Martinez et al., 2018). The current data demonstrate that the NAcc is a key node in all three behavioral paradigms (Figure 5). In the FAM network for saline-treated animals, NAcc coordinated activity is enhanced by OTA but knocked out by OT treatment. In the UNFAM network for saline-treated animals, the NAcc is the key active node out of the mesolimbic reward circuit and is knocked out by both OT and OTA. Finally, in the PUP network for saline-treated animals, all mesolimbic reward circuit regions are key nodes. This includes the NAcc, which is then knocked out to varying extents by OT and OTA treatment. Given its role in assigning valence to social stimuli, it will be important to evaluate OT effects directly in the NAcc to better understand how this region alters coordinated neural activity, similar to Johnson et al. (2017), across different social contexts.

We have taken a broad approach in our first attempt at teasing apart function in the naked mole-rat SDMN. For this reason, we did not break down groups by subcaste or focus on a single sex for stimulus animal. Subordinates are pre-pubertal, do not exhibit sex differences in behavior (Lacey and Sherman, 1991), and take a week to begin demonstrating behaviors characteristic of reproductive maturation (Margulis et al., 1995; Clarke and Faulkes, 1997; Mooney et al., 2015b; Swift-Gallant et al., 2015; Toor et al., 2015). Thus, it is likely that the sex of stimulus animals would cause a negligible effect given the very short period of removal from colony. It, of course, also warrants mention that our experimental design was likely insufficiently powered to robustly examine OT manipulation by behavioral paradigm interactions. Our sample size was limited by the challenges of rearing naked mole-rats in captivity (taking 1 year to reach adulthood coupled with unpredictable breeding). Low statistical power might also explain why we did not detect any significant effects of OT manipulation on behavior or significant relationships between c-Fos activity and behavior, though the lack of behavioral results following central administration of OT is not necessarily unexpected. While we have previously shown that peripheral OT administration changes in-colony behavior (e.g., time spent huddling) and proximity to novel conspecifics, we did not measure in-colony behavior in the current study and also used a different testing apparatus for outpairing (single chamber vs. double chamber) (Mooney et al., 2014). To address these limitations, we provide the raw data in Supplementary Information with the hope that future work by us and others will build on this sample.

Here we report the first formal investigation of activation of the SDMN in the highly social naked mole-rat. We have demonstrated that coordinated neural activity within this network and with related regions varies according to social context, and that this coordinated activation is altered by manipulation of the OT system. The pattern of connectivity associated with each behavioral paradigm suggests a role for the NAcc in social valence and sociosexual interaction, the mPFC in assessing/establishing social dominance, and the hippocampus in pup recognition.

## Author Contributions

MH and SM contributed conception and design of the study. SM and MF-M collected the data. MF-M performed statistical analyses and wrote the first draft of the manuscript. SM performed the experiments and wrote sections of the manuscript. All authors contributed to manuscript revision, read, and approved the submitted version.

## Conflict of Interest Statement

The authors declare that the research was conducted in the absence of any commercial or financial relationships that could be construed as a potential conflict of interest.

## Abbreviations

ACC: anterior cingulate cortex
AH: anterior hypothalamus
AntPVN: anterior paraventricular nucleus
AON: anterior olfactory nucleus
BLA: basolateral amygdala
BNST: bed nucleus of the stria terminalis
CA1: cornu ammonis 1
CA2: cornu ammonis 2
CA3: cornu ammonis 3
Caudate: caudate putamen
CeA: central amygdala
CG: cingulate cortex
dDG: dorsal dentate gyrus
IL: infralimbic cortex
LS: lateral septum
MeA: medial amygdala
MOB: main olfactory bulb
MS: medial septum
NAcc: nucleus accumbens
PG: periaqueductal gray
PIC: piriform cortex
PO: pre-optic area
PostPVN: posterior paraventricular nucleus
PrL: pre-limbic cortex
SON: supraoptic nucleus
Tu: olfactory tubercle
vDG: ventral dentate gyrus
VMH: ventromedial hypothalamus
VP: ventral pallidum
VTA: ventral tegmental area.

## References

[B1] Anacker A. M. J. Beery A. K. (2013). Life in groups: the roles of oxytocin in mammalian sociality. *Front. Behav. Neurosci.* 7:185. 10.3389/fnbeh.2013.00185 24376404 PMC3858648

[B2] Barrett C. E. Keebaugh A. C. Ahern T. H. Bass C. E. Terwilliger E. F. Young L. J. (2013). Variation in vasopressin receptor (Avpr1a) expression creates diversity in behaviors related to monogamy in prairie voles. *Horm. Behav.* 63 518–526. 10.1016/j.yhbeh.2013.01.005 23370363 PMC3602142

[B3] Beery A. K. Bicks L. Mooney S. J. Goodwin N. L. Holmes M. M. (2016). Sex, social status, and CRF receptor densities in naked mole-rats. *J. Comp. Neurol.* 524 228–243. 10.1002/cne.23834 26100759

[B4] Beery A. K. Lacey E. A. Francis D. D. (2008). Oxytocin and vasopressin receptor distributions in a solitary and a social species of tuco-tuco (*Ctenomys haigi* and *Ctenomys sociabilis*). *J. Comp. Neurol.* 507 1847–1859. 10.1002/cne.21638 18271022

[B5] Bicks L. K. Koike H. Akbarian S. Morishita H. (2015). Prefrontal cortex and social cognition in mouse and man. *Front. Psychol.* 6:1805. 10.3389/fpsyg.2015.01805 26635701 PMC4659895

[B6] Brennan P. A. Kendrick K. M. (2006). Mammalian social odours: attraction and individual recognition. *Philos. Trans. R. Soc. B Biol. Sci.* 361 2016–2078. 10.1098/rstb.2006.1931 17118924 PMC1764843

[B7] Brett R. A. (1991a). “The ecology of naked mole-rat colonies: burrowing, food, and limiting factors.(pepper braude lacey Sherman 1991),” in *The Biology of the Naked Mole-Rat*, eds Sherman P. W. Jarvis J. U. M. Alexander R. D. (Princeton: Princeton University Press), 137–184.

[B8] Brett R. A. (1991b). “The population structure of naked mole-rat colonies,” in *The Biology of the Naked Mole-Rat*, eds Sherman P. W. Jarvis J. U. M. Alexander R. D. (Princeton: Princeton University Press), 97–136.

[B9] Champagne F. Diorio J. Sharma S. Meaney M. J. (2001). Naturally occurring variations in maternal behavior in the rat are associated with differences in estrogen-inducible central oxytocin receptors. *Proc. Natl. Acad. Sci. U.S.A.* 98 12736–12741. 10.1073/pnas.221224598 11606726 PMC60123

[B10] Chang S. W. C. Fagan N. A. Toda K. Utevsky A. V. Pearson J. M. Platt M. L. (2015). Neural mechanisms of social decision-making in the primate amygdala. *Proc. Natl. Acad. Sci. U.S.A.* 112 16012–16017. 10.1073/pnas.1514761112 26668400 PMC4702988

[B11] Choleris E. Little S. R. Mong J. A. Puram S. V. Langer R. Pfaff D. W. (2007). Microparticle-based delivery of oxytocin receptor antisense DNA in the medial amygdala blocks social recognition in female mice. *Proc. Natl. Acad. Sci. U.S.A.* 104 4670–4675. 10.1073/pnas.0700670104 17360582 PMC1838659

[B12] Clarke F. M. Faulkes C. G. (1997). Dominance and queen succession in captive colonies of the eusocial naked mole-rat, *Heterocephalus glaber*. *Proc. Biol. Sci.* 264 993–1000. 10.1098/rspb.1997.0137 9263466 PMC1688532

[B13] Consiglio A. R. Borsoi A. Pereira G. A. M. Lucion A. B. (2005). Effects of oxytocin microinjected into the central amygdaloid nucleus and bed nucleus of stria terminalis on maternal aggressive behavior in rats. *Physiol. Behav.* 85 354–362. 10.1016/j.physbeh.2005.05.002 15935410

[B14] Curtis J. T. Liu Y. Wang Z. (2001). Lesions of the vomeronasal organ disrupt mating-induced pair bonding in female prairie voles (*Microtus ochrogaster*). *Brain Res.* 901 167–174. 10.1016/S0006-8993(01)02343-5 11368964

[B15] Faykoo-Martinez M. Ashley Monks D. Zovkic I. B. Holmes M. M. (2018). Sex- and brain region-specific patterns of gene expression associated with socially-mediated puberty in a eusocial mammal. *PLoS One* 13:e0193417. 10.1371/journal.pone.0193417 29474488 PMC5825099

[B16] Ferguson J. N. Aldag J. M. Insel T. R. Young L. J. (2001). Oxytocin in the medial amygdala is essential for social recognition in the mouse. *J. Neurosci.* 21 8278–8285. 10.1523/JNEUROSCI.21-20-08278.2001 11588199 PMC6763861

[B17] Ferguson J. N. Young L. J. Hearn E. F. Matzuk M. M. Insel T. R. Winslow J. T. (2000). Social amnesia in mice lacking the oxytocin gene. *Nat. Genet.* 25 284–288. 10.1038/77040 10888874

[B18] Francis D. D. Champagne F. C. Meaney M. J. (2000). Variations in maternal behaviour are associated with differences in oxytocin receptor levels in the rat. *J. Neuroendocrinol.* 12 1145–1148. 10.1046/j.1365-2826.2000.00599.x 11106970

[B19] Geissmann Q. (2013). OpenCFU, a new free and open-source software to count cell colonies and other circular objects. *PLoS One* 8:e50472. 10.1371/journal.pone.0054072 23457446 PMC3574151

[B20] Goodson J. L. Kabelik D. (2009). Dynamic limbic networks and social diversity in vertebrates: from neural context to neuromodulatory patterning. *Front. Neuroendocrinol.* 30 429–441. 10.1016/j.yfrne.2009.05.007 19520105 PMC2763925

[B21] Harrell F. E. (2017). *CRAN – Package Hmisc. Hmisc Harrell Misc.* Available at: http://biostat.mc.vanderbilt.edu/Hmisc

[B22] Hathaway G. A. Faykoo-Martinez M. Peragine D. E. Mooney S. J. Holmes M. M. (2016). Subcaste differences in neural activation suggest a prosocial role for oxytocin in eusocial naked mole-rats. *Horm. Behav.* 79 1–7. 10.1016/j.yhbeh.2015.12.001 26718226

[B23] Holmes M. M. Van Mil S. Bulkowski C. Goldman S. L. Goldman B. D. Forger N. G. (2013). Androgen receptor distribution in the social decision-making network of eusocial naked mole-rats. *Behav. Brain Res.* 256 214–218. 10.1016/j.bbr.2013.08.025 23973387

[B24] Hyer M. M. Hunter T. J. Katakam J. Wolz T. Glasper E. R. (2016). Neurogenesis and anxiety-like behavior in male California mice during the mate’s postpartum period. *Eur. J. Neurosci.* 43 703–709. 10.1111/ejn.13168 26750200

[B25] IBM Corp (2016). *IBM SPSS Statistics for Macintosh, Version 24.0.*

[B26] Jarvis J. U. M. (1981). Eusociality in a mammal: cooperative breeding in naked mole-rat colonies. *Science* 212 571–573. 10.1126/science.7209555 7209555

[B27] Johnson Z. V. Walum H. Jamal Y. A. Xiao Y. Keebaugh A. C. Inoue K. (2016). Central oxytocin receptors mediate mating-induced partner preferences and enhance correlated activation across forebrain nuclei in male prairie voles. *Horm. Behav.* 79 8–17. 10.1016/j.yhbeh.2015.11.011 26643557 PMC4768463

[B28] Johnson Z. V. Walum H. Xiao Y. Riefkohl P. C. Young L. J. (2017). Oxytocin receptors modulate a social salience neural network in male prairie voles. *Horm. Behav.* 87 16–24. 10.1016/j.yhbeh.2016.10.009 27793769 PMC5207344

[B29] Kalamatianos T. Faulkes C. G. Oosthuizen M. K. Poorun R. Bennett N. C. Coen C. W. (2010). Telencephalic binding sites for oxytocin and social organization: â comparative study of eusocial naked mole-rats and solitary cape mole-rats. *J. Comp. Neurol.* 518 1792–1813. 10.1002/cne.22302 20235093

[B30] Kinsley C. H. Madonia L. Gifford G. W. Tureski K. Griffin G. R. Lowry C. (1999). Motherhood improves learning and memory. *Nature* 402 137–138. 10.1038/45957 10647003

[B31] Kuznetsova A. Brockhoff P. Christensen R. (2016). *lmerTest: Tests in Linear Mixed Effects Models. R Packag. version.* 10.18637/jss.v082.i13

[B32] Lacey E. A. Sherman P. W. (1991). “Social organization of naked mole-rat colonies: evidence for divisions of labor,” in *The Biology of the Naked Mole-Rat*, eds Sherman P. W. Jarvis J. U. M. Alexander R. D. (Princeton: Princeton University Press).

[B33] Lee E. Rhim I. Lee J. W. Ghim J.-W. Lee S. Kim E. (2016). Enhanced neuronal activity in the medial prefrontal cortex during social approach behavior. *J. Neurosci.* 36 6926–6936. 10.1523/jneurosci.0307-16.2016 27358451 PMC6604896

[B34] Lee V. K. Harris L. T. (2013). How social cognition can inform social decision making. *Front. Neurosci.* 7:259 10.3389/fnins.2013.00259 PMC3872305 24399928

[B35] Lim M. M. Young L. J. (2004). Vasopressin-dependent neural circuits underlying pair bond formation in the monogamous prairie vole. *Neuroscience* 125 35–45. 10.1016/j.neuroscience.2003.12.008 15051143

[B36] Margulis S. W. Saltzman W. Abbott D. H. (1995). Behavioral and hormonal changes in female naked mole-rats (*Heterocephalus glaber*) following removal of the breeding female from a colony. *Horm. Behav.* 29 227–247. 10.1006/hbeh.1995.1017 7557925

[B37] McCann K. E. Rosenhauer A. M. Jones G. M. F. Norvelle A. Choi D. C. Huhman K. L. (2017). Histone deacetylase and acetyltransferase inhibitors modulate behavioral responses to social stress. *Psychoneuroendocrinology* 75 100–109. 10.1016/j.psyneuen.2016.10.022 27810703 PMC5135625

[B38] Mooney S. J. Coen C. W. Holmes M. M. Beery A. K. (2015a). Region-specific associations between sex, social status, and oxytocin receptor density in the brains of eusocial rodents. *Neuroscience* 303 261–269. 10.1016/j.neuroscience.2015.06.043 26143015

[B39] Mooney S. J. Filice D. C. S. Douglas N. R. Holmes M. M. (2015b). Task specialization and task switching in eusocial mammals. *Anim. Behav.* 109 227–233. 10.1016/j.anbehav.2015.08.019

[B40] Mooney S. J. Douglas N. R. Holmes M. M. (2014). Peripheral administration of oxytocin increases social affiliation in the naked mole-rat (*Heterocephalus glaber*). *Horm. Behav.* 65 380–385. 10.1016/j.yhbeh.2014.02.003 24530845

[B41] Mooney S. J. Holmes M. M. (2013). Social condition and oxytocin neuron number in the hypothalamus of naked mole-rats (*Heterocephalus glaber*). *Neuroscience* 230 56–61. 10.1016/j.neuroscience.2012.11.014 23200787

[B42] Mooney S. J. Holmes M. M. (2015). Successful intracerebroventricular cannulation of a eusocial mammal. *J. Neurosci. Methods* 239 75–79. 10.1016/j.jneumeth.2014.09.026 25285986

[B43] Nakajima M. Görlich A. Heintz N. (2014). Oxytocin modulates female sociosexual behavior through a specific class of prefrontal cortical interneurons. *Cell* 159 295–305. 10.1016/j.cell.2014.09.020 25303526 PMC4206218

[B44] Newman S. (1999). The medial extended amygdala in male reproductive behavior. *Ann. N. Y. Acad. Sci.* 877 242–257. 10.1111/j.1749-6632.1999.tb09271.x 10415653

[B45] O’Connell L. A. Hofmann H. A. (2011). The vertebrate mesolimbic reward system and social behavior network: a comparative synthesis. *J. Comp. Neurol.* 519 3599–3639. 10.1002/cne.22735 21800319

[B46] O’Connell L. A. Hofmann H. A. (2012). Evolution of a vertebrate social decision-making network. *Science* 336 1154–1157. 10.1126/science.1218889 22654056

[B47] Oettl L. L. Ravi N. Schneider M. Scheller M. F. Schneider P. Mitre M. (2016). Oxytocin enhances social recognition by modulating cortical control of early olfactory processing. *Neuron* 90 609–621. 10.1016/j.neuron.2016.03.033 27112498 PMC4860033

[B48] Olazábal D. E. Young L. J. (2006a). Oxytocin receptors in the nucleus accumbens facilitate “spontaneous” maternal behavior in adult female prairie voles. *Neuroscience* 141 559–568. 10.1016/j.neuroscience.2006.04.017 16725274

[B49] Olazábal D. E. Young L. J. (2006b). Species and individual differences in juvenile female alloparental care are associated with oxytocin receptor density in the striatum and the lateral septum. *Horm. Behav.* 49 681–687. 10.1016/j.yhbeh.2005.12.010 16442534

[B50] Pepper J. W. Braude S. H. Lacey E. A. Sherman P. W. (1991). “Vocalizations of the naked mole-rat,” in *The Biology of the Naked Mole-Rat*, eds Sherman P. W. Jarvis J. U. M. Alexander R. D. (Princeton: Princeton University Press), 243–274.

[B51] Pinheiro J. Bates D. DebRoy S. Sarkar D. (2016). *nlme: Linear and Nonlinear Mixed Effects Models. R Packag. version.*

[B52] R Development Core Team (2011). *R: A Language and Environment for Statistical Computing.* Vienna: R Foundation for Statistical Computing.

[B53] Rasband W. (2012). *ImageJ.* Bethesda, MD: U.S. National Institutes of Health.

[B54] Reddon A. R. Voisin M. R. O’Connor C. M. Balshine S. (2014). Isotocin and sociality in the cooperatively breeding cichlid fish, *Neolamprologus pulcher*. *Behaviour* 151 1389–1411. 10.1163/1568539X-00003190

[B55] Rosen G. J. De Vries G. J. Goldman S. L. Goldman B. D. Forger N. G. (2007). Distribution of vasopressin in the brain of the eusocial naked mole-rat. *J. Comp. Neurol.* 500 1093–1105. 10.1002/cne.21215 17183541

[B56] Rosen G. J. de Vries G. J. Goldman S. L. Goldman B. D. Forger N. G. (2008). Distribution of oxytocin in the brain of a eusocial rodent. *Neuroscience* 155 809–817. 10.1016/j.neuroscience.2008.05.039 18582538 PMC2614305

[B57] Ruscio M. G. Sweeny T. D. Hazelton J. L. Suppatkul P. Boothe E. Carter C. S. (2008). Pup exposure elicits hippocampal cell proliferation in the prairie vole. *Behav. Brain Res.* 187 9–16. 10.1016/j.bbr.2007.08.028 17913255 PMC2699755

[B58] Samuelsen C. L. Meredith M. (2011). Oxytocin antagonist disrupts male mouse medial amygdala response to chemical-communication signals. *Neuroscience* 180 96–104. 10.1016/j.neuroscience.2011.02.030 21333718 PMC3093756

[B59] Shahrokh D. K. Zhang T. Y. Diorio J. Gratton A. Meaney M. J. (2010). Oxytocin-dopamine interactions mediate variations in maternal behavior in the rat. *Endocrinology* 151 2276–2286. 10.1210/en.2009-1271 20228171 PMC2869254

[B60] Shannon P. Markiel A. Owen Ozier O. Baliga N. S. Wang J. T. Ramage D. (2003). Cytoscape: a software environment for integrated models of biomolecular interaction networks. *Genome Res.* 13 2498–2504. 10.1101/gr.1239303.metabolite 14597658 PMC403769

[B61] Swift-Gallant A. Mo K. Peragine D. E. Monks D. A. Holmes M. M. (2015). Removal of reproductive suppression reveals latent sex differences in brain steroid hormone receptors in naked mole-rats, *Heterocephalus glaber*. *Biol. Sex Differ.* 6:31. 10.1186/s13293-015-0050-x 26693002 PMC4676092

[B62] Takahashi A. Nagayasu K. Nishitani N. Kaneko S. Koide T. (2014). Control of intermale aggression by medial prefrontal cortex activation in the mouse. *PLoS One* 9:e94657. 10.1371/journal.pone.0094657 24740241 PMC3989250

[B63] Teles M. C. Almeida O. Lopes J. S. Oliveira R. F. (2015). Social interactions elicit rapid shifts in functional connectivity in the social decision-making network of zebrafish. *Proc. R. Soc. B Biol. Sci.* 282:20151099. 10.1098/rspb.2015.1099 26423839 PMC4614767

[B64] Tobin V. A. Hashimoto H. Wacker D. W. Takayanagi Y. Langnaese K. Caquineau C. (2010). An intrinsic vasopressin system in the olfactory bulb is involved in social recognition. *Nature* 464 413–417. 10.1038/nature08826 20182426 PMC2842245

[B65] Toor I. Clement D. Carlson E. N. Holmes M. M. (2015). Olfaction and social cognition in eusocial naked mole-rats, *Heterocephalus glaber*. *Anim. Behav.* 107 175–181. 10.1016/j.anbehav.2015.06.015

[B66] Wang F. Zhu J. Zhu H. Zhang Q. Lin Z. Hu H. (2011). Bidirectional control of social hierarchy by synaptic efficacy in medial prefrontal cortex. *Science* 334 693–697. 10.1126/science.1209951 21960531

[B67] Wei T. Simko V. (2016). *The Corrplot package.* Vienna: R Foundation for Statistical Computing.

[B68] Williamson C. M. Klein I. S. Lee W. Curley J. P. (2018). Immediate early gene activation throughout the brain is associated with dynamic changes in social context. *Soc. Neurosci.* 31 1–13. 10.1101/275495 29781376

[B69] Withers P. C. Jarvis J. U. M. (1980). The effect of huddling on thermoregulation and oxygen consumption for the naked mole-rat. *Comp. Biochem. Physiol. Part A Physiol.* 66 215–219. 10.1016/0300-9629(80)90154-1

[B70] Zheng D. J. Larsson B. Phelps S. M. Ophir A. G. (2013). Female alternative mating tactics, reproductive success and nonapeptide receptor expression in the social decision-making network. *Behav. Brain Res.* 246 139–147. 10.1016/j.bbr.2013.02.024 23500897 PMC3633648

