# Supplementary material for: Oxytocin Manipulation Alters Neural Activity in Response to Social Stimuli in Eusocial Naked Mole-Rats
Source: Front Behav Neurosci. 2018 Nov 20;12:272. doi: 10.3389/fnbeh.2018.00272 (PMC6255855; doi:10.3389/fnbeh.2018.00272)
Supplement: Supplementary file 1 [file Table_1.DOCX]

**Supplementary Table 1**. Linear mixed effects model results comparing change in behavior duration from baseline test to collection day (where oxytocin was manipulated).

| Behavior type | Main effect of OT manipulation | Main effect of sex | OT manipulation-by-sex interaction |
| --- | --- | --- | --- |
| Animals exposed to familiar conspecific (FAM) | | | |
| Aggression | F(2,15)=0.14, p=0.86 | F(1,15)=1.06, p=0.32 | F(2,15)=1.94, p=0.18 |
| Anogenital Investigation | F(2,15)=0.78, p=0.48 | F(1,15)=0.92, p=0.35 | F(2,15)=0.59, p=0.56 |
| Investigation (Flank/Face) | F(2,15)=3,73, p=0.05 | F(1,15)=0.18, p=0.67 | F(2,15)=1.71, p=0.21 |
| Animals exposed to unfamiliar conspecific (UNFAM) | | | |
| Aggression | F(2,24)=0.19 p=0.83 | F(1,24)=2.41, p=0.13 | F(2,24)=0.18, p=0.84 |
| Anogenital Investigation | F(2,24)=0.27, p=0.76 | F(1,24)=0.09, p=0.77 | F(2,24)=1.36, p=0.27 |
| Investigation (Flank/Face) | F(2,24)=2.56, p=0.10 | F(1,24)=1.48, p=0.24 | F(2,24)=0.71, p=0.50 |
| Animals exposed to pups (PUP) | | | |
| Pup carrying | F(2,18)=0.12, p=0.88 | F(1,18)=0.09, p=0.76 | F(2,18)=1.72, p=0.21 |
| Pup investigation | F(2,18)=0.59, p=0.56 | F(1,18)=0.74, p=0.40 | F(2,18)=0.34, p=0.72 |
