# Supplementary material for: Oxytocin Manipulation Alters Neural Activity in Response to Social Stimuli in Eusocial Naked Mole-Rats
Source: Front Behav Neurosci. 2018 Nov 20;12:272. doi: 10.3389/fnbeh.2018.00272 (PMC6255855; doi:10.3389/fnbeh.2018.00272)
Supplement: Supplementary file 2 [file Table_2.DOCX]

**Supplementary Table 2**. Linear mixed effects model results for behavior data on collection day with OT manipulation and sex as independent variables. Behavior is measured as duration.

| Behavior type | Main effect of OT manipulation | Main effect of sex | OT manipulation-by-sex interaction |
| --- | --- | --- | --- |
| Animals exposed to familiar conspecific (FAM) | | | |
| Aggression | F(2,11)=0.26, p=0.78 | F(1,11)=0.78, p=0.40 | F(2,11)=2.07, p=0.17 |
| Anogenital Investigation | F(2,11)=2.46, p=0.13 | F(1,11)=0.05, p=0.83 | F(2,11)=1.14, p=0.35 |
| Investigation (Flank/Face) | F(2,14)=1.72, p=0.21 | F(1,14)=0.03, p=0.85 | F(2,14)=0.11, p=0.89 |
| Animals exposed to unfamiliar conspecific (UNFAM) | | | |
| Aggression | F(2,24)=1.84, p=0.18 | F(1,24)=1.57, p=0.22 | F(2,24)=2.08, p=0.15 |
| Anogenital Investigation | F(2,24)=1.86, p=0.18 | F(1,24)=0.09, p=0.76 | F(2,24)=0.69, p=0.51 |
| Investigation (Flank/Face) | F(2,23)=3.05, p=0.67 | F(1,23)=2.62, p=0.12 | F(2,23)=0.35, p=0.71 |
| Animals exposed to pups (PUP) | | | |
| Pup carrying | F(2,18)=0.07, p=0.93 | F(1,18)=0.28, p=0.60 | F(2,18)=1.28, p=0.30 |
| Pup interaction | F(2,18)=0.59, p=0.56 | F(1,18)=0.00, p=0.95 | F(2,18)=1.78, p=0.20 |
